# On the Origins of Toughness in *Corymbia calophylla* (Marri Tree) Nuts

**DOI:** 10.1002/advs.202515273

**Published:** 2026-03-25

**Authors:** Wegood M. Awad, Ahmed S. Dalaq, Marieh B. Al‐Handawi, Rezo Getsadze, Oraib Al‐Ketan, James Weston, Mohammed Daqaq, Panče Naumov

**Affiliations:** ^1^ Smart Materials Lab New York University Abu Dhabi Abu Dhabi UAE; ^2^ Center for Translational Medical Devices, NYUAD Research Institute New York University Abu Dhabi Abu Dhabi UAE; ^3^ Center for Smart Engineering Materials, NYUAD Research Institute New York University Abu Dhabi Abu Dhabi UAE; ^4^ Bioengineering Department King Fahd University of Petroleum & Minerals Dhahran Saudi Arabia; ^5^ Interdisciplinary Research Center (IRC) for Biosystems and Machines King Fahd University of Petroleum & Minerals Dhahran Saudi Arabia; ^6^ Informatics Institute Faculty of Science University of Amsterdam Amsterdam Netherlands; ^7^ Core Technology Platforms New York University Abu Dhabi Abu Dhabi UAE; ^8^ Division of Engineering New York University Abu Dhabi Abu Dhabi UAE; ^9^ Department of Mechanical Engineering Tandon School of Engineering New York University New York USA; ^10^ Research Center for Environment and Materials Macedonian Academy of Sciences and Arts Skopje Macedonia; ^11^ Molecular Design Institute, Department of Chemistry New York University New York USA

**Keywords:** biocomposites, biomimetics, digital image correlation, marri, mechanical properties, nuts

## Abstract

Fruits and nuts develop resilient outer shells to safeguard their embryos against environmental hazards and predators. The nuts of the marri tree (*Corymbia calophylla*) possess unusual strength and toughness. In contrast to other tough nuts, seasoned parrots avoid cracking them and instead resort to drawn‐out attempts to snatch their seeds from a dispersal opening at their base. Here, we reveal the subtle strengthening mechanisms of the marri nut and compare its mechanical response and properties to those of other nuts and commercial materials. The analysis involves an extensive examination of the various material phases of the nutshell, the chemical composition of its fibers, and an analysis of its microscopic and macroscopic mechanical properties. The study reveals that, akin to self‐standing structures, the interplay of the densely packed fibers of the shell held together by a highly ductile soft matrix, while arranged in a spherical configuration, gives the nut its notable toughness, ductility, strength, and remarkable fracture resistance. Additionally, we design and test the mechanical behavior of a bioinspired interpenetrating phase composite material mimicking the nut's microstructure, demonstrating comparable deformability and toughness. These findings lay a new foundation for designing highly efficient bioinspired structures for energy absorption, protection, and armor.

## Graphical abstract

We uncover the natural toughening mechanisms of the marri nut, including fiber pullout, crack deflection, and a viscoelastic matrix, which enable exceptional energy absorption and ductility comparable to Teflon, with an elastic modulus similar to acrylic. Inspired by this, we engineered a 3D printed interpenetrating composite mimicking the nut's structure, aiming to replicate these properties for protective applications and energy‐absorbing structures.

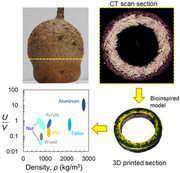

## 1 Introduction

**FIGURE 1:**
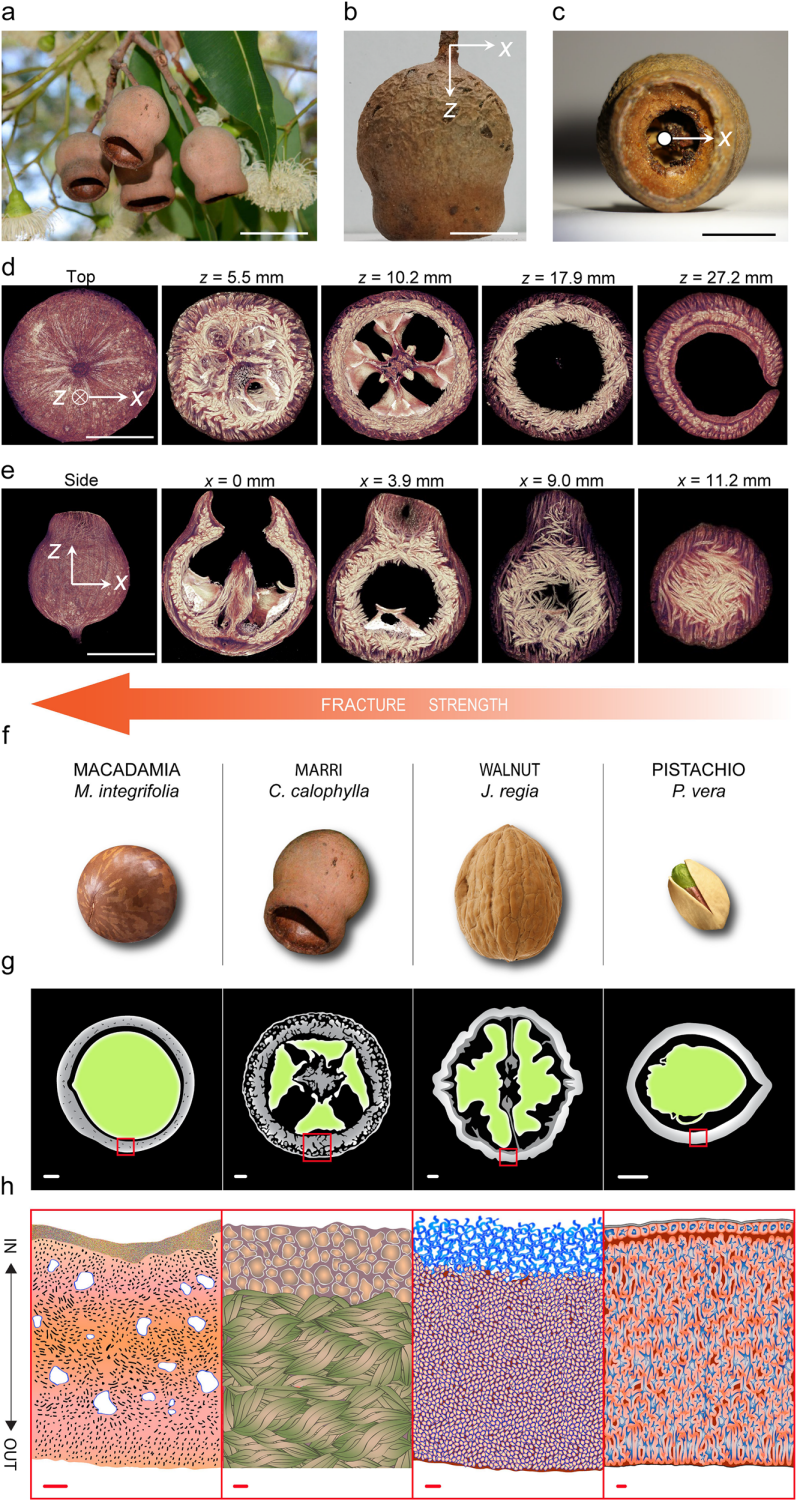
Marri nut structure and dimensions, and comparison with other nuts. (a–c) Nut (a) hanging from its mother tree, and (b) side view, and (c) front view of the nut. (d,e) Cross‐sectional slices of the CT scan shown from the top view at *z* = 5.5, 10.2, 17.9, and 27.2 mm (d), and from the side view at *x* = 0, 3.9, 9.0, and 11.2 mm (e). (f–h) Schematic showing the optical (f), CT (g), and microscopic (h) images of the different nut microstructures. Scale bars are 10 mm (a–e), 2 mm (g), and 100 µm (h). Image 1a was recorded by D. Blumer [9] and is reproduced with permission of the Botanic Gardens and Parks Authority. The graph in panel h was adapted from Ref. [8].

In the current academic and industrial landscapes, there is a growing urgency to discover novel materials that transcend the limitations of traditional materials such as steel, acrylic, and wood. One approach draws upon Nature's refined design principles over millions of years to prepare bioinspired materials with all‐new and unique properties. Chemical and mechanistic analyses of natural systems renowned for energy absorption, puncture resistance, and purification have started to unveil a promising pathway toward designing bioinspired materials for energy absorption, armors, cushion materials, and protection devices [1, 2]. For instance, innovative water purification methods have emerged inspired by the selective water uptake and nutrient removal abilities of plant roots [3]. Additionally, using natural fibers from bamboo, hemp, and flax has played a pivotal role in creating rigid fiber composites [4], with each fiber type providing unique properties to the reinforced materials. Insights drawn from fruits like pomelo [5] and durian [6] have provided guidelines for designing energy absorption materials, such as foams and hierarchical structures [7], which exhibit low density, high strength, and considerable energy absorption capacities. Due to their remarkable mechanical properties, such as strength, toughness, and hardness, nuts have also recently caught attention as an inspiring source for biomimicry. Their desirable properties arise from different strengthening mechanisms, some of which still need to be fully understood [8]. For example, the protective shells of various nuts, including almonds, macadamias, and walnuts, demonstrate high toughness and puncture resistance against natural predators and environmental conditions. The nuts of *Corymbia calophylla*, a plant native to Western Australia [9] that is locally known as the marri tree, have especially garnered our attention because of their unique resistance to cracking by parrots and other predatory birds (Figure 1a) [10]. Although parrots can employ precise techniques using their sharp beaks and formidable jaw strength, capable of exerting forces of up to 75 N on a relatively small surface area [11], they are unable to penetrate marri nuts. Instead, they often resort to persistent attempts to extract the seeds from the seed's dispersal opening located at its base. This fascinating resilience and outwitting of the predators suggest that the nut may possess unique biological, chemical, or structural features yielding mechanical strategies that make it unusually robust. In this study, we explore the structural attributes and mechanical properties of the marri nut through tomography, indentation, spectroscopy, alignment vector analysis, and ring‐compression tests. Using these tools, we investigate the microscopic structure and macro‐mechanical properties of the nut, including its apparent elastic modulus, overall toughness, and deformability, and then compare them to other commercially available materials. Findings reveal superior energy absorption per unit volume (toughness) that may be attributed to its microstructure, which is composed of highly intertwined fibers and multiphase interpenetrating composites made of stiff fibers and soft matrix material. Inspired by these findings, we engineered a composite material fabricated using two contrasting materials that mimic the natural nut's soft matrix and stiff fibers. Mechanical testing of the engineered material demonstrated high toughness, highlighting its potential as a bioinspired material for the design of ultra‐tough armor, protective devices, sportswear, tires, greenhouse covers, and medical gloves.

## 2 Results and Discussion

### 2.1 General Features of the Marri Nut

The nut has a bell‐shaped shell, approximately 5 mm thick, with a diameter of around 25 mm and a height of about 29 mm, hanging from the tree via a stem with a diameter of about 3 mm (Figure 1b). The seeds are found inside this hollow bell, enclosed within the seed pockets, and are released for reproductive purposes through a dispersal hole (Figure 1c). Cross‐sectional views obtained along the height of the nut (*z*‐direction) using computed tomography (CT) reveal that the nut houses its seeds within four distinct pockets (Figure 1c). The pockets, divided by relatively less dense fiber‐based walls, start tight near the stem (*z* = 5.5 mm) and widen toward the center (*z* = 10 mm). For *z* > 16 mm, a partially separated section appears, marking the beginning of the nut's seed dispersal opening (0.35 times the diameter of the nut). The nutshell is divided into two regions: a dark region forming the outer layer of the nut, and a relatively thicker, light brown inner layer (Figure 1d). Side cross‐sectional views obtained along the width of the nut (*x*‐direction) reveal an intricate structure composed of dense fibers originating from the stem, which protrude along the *z*‐axis inside the nut cavity (*x* = 0 mm) (Figure 1e). Physical inspection of this protruded support shows that it is exceptionally stiff and challenging to cut; thus, it acts as a stud through which fibers grow, intertwine, and finally lignify. The difference in the color gradation of the CT scans may indicate the gradation of the fiber's density and general material composition across the nut. Figure 1e, in general, and specifically at *x* = 11.2 mm, reveals that the bundles of fibers forming the nut are highly directional, implying significant anisotropy in its physical and mechanical properties. The marri nut's anisotropic, fiber‐based architecture sets it apart as one of only two known examples of fiber reinforcement in nuts, alongside macadamia [8] and Brazil nut [12, 13]. In contrast, many other nuts rely on interlocking networks of irregularly shaped cells, such as those observed in pistachio [8] and walnut [14]. A schematic comparison of these three well‐studied nuts with the marri nut is shown in Figure 1, panels f–h. The force required to initiate cracking across different nut types is provided in the Figures S1 and S2, where the marri nut ranks second among the analyzed nuts.

### 2.2 Structural Features and Constituents

**FIGURE 2:**
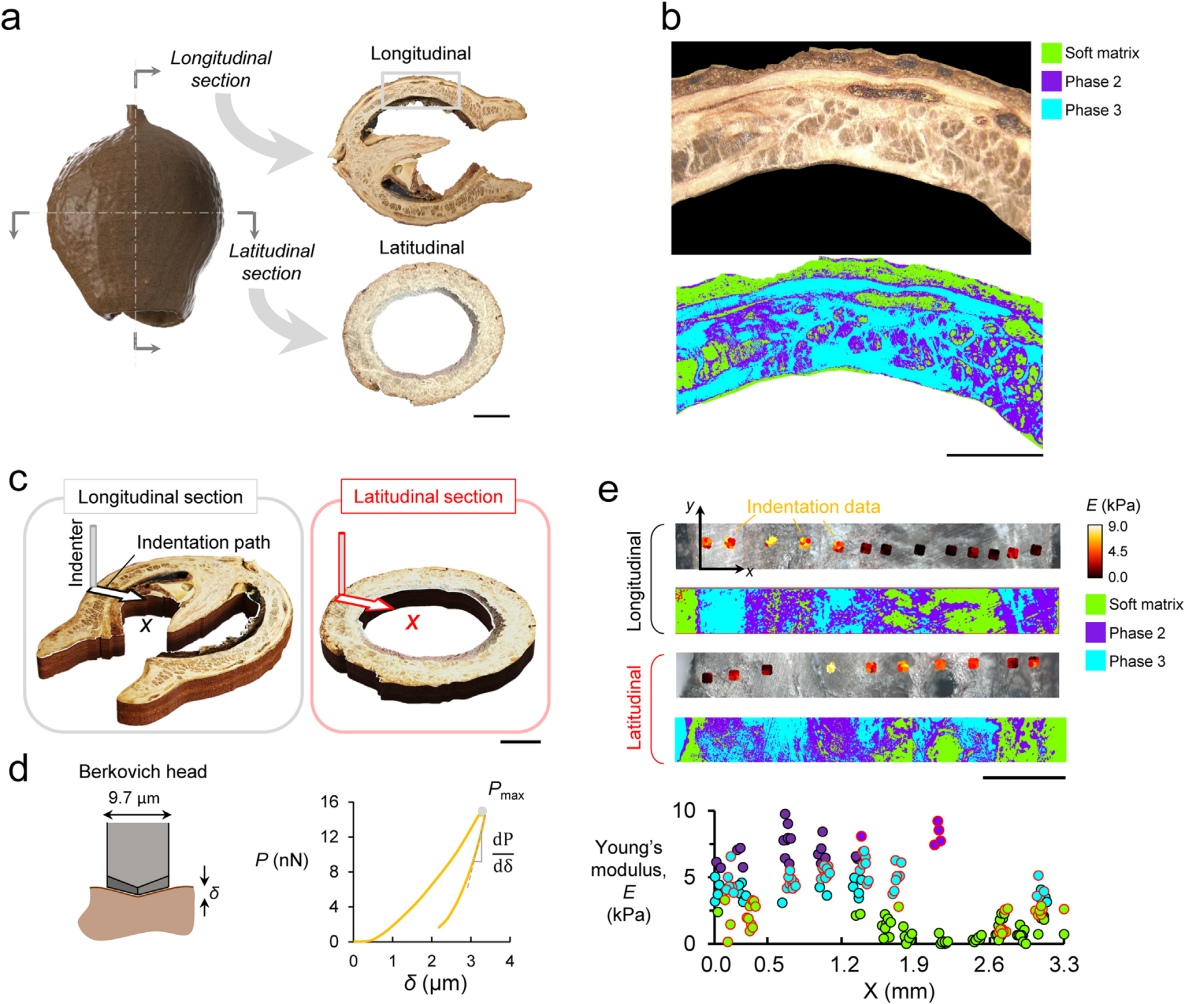
Structural features and localized properties of the marri nut. (a) Voxel‐based reconstruction of the nut, along with actual cuts performed along the longitudinal and latitudinal sections. (b) Microscopic image of the surface along the longitudinal plane together with the segmented image using *k*‐means clustering. (c) The direction of the serial nanoindentation performed in longitudinal and latitudinal sections. The scale bars in panels (a−c) are 5 mm. (d) Size and shape of the Berkovich head used in the indentation procedure, along with a representative *P*−*δ* curve. (e) Elastic modulus measurement with its corresponding segmented regions and respective phase designations. The scale bar in panel e is 1 mm.

The previously obtained CT scan allows the reconstruction of a 3D voxel‐based model of the nut, as shown in Figure 2a. To achieve this, longitudinal and latitudinal cuts were acquired using a high‐precision saw. Figure 2a shows two sections of an actual sample, exposing the seed pocket and the central stud at the root of the stem. Using an optical microscope, a high‐definition surface image of a smaller window was obtained along the plane of the longitudinal section (Figure 2b). This high‐definition image was further post‐processed using the *k*‐means clustering algorithm, which systematically segments the images into distinct regions (for details, see the Methods section). Figure 2b depicts the microscopic image segmented into three distinct regions, referred to as phases 1, 2, and 3. We suggest that the first phase could be the soft matrix due to its distinct color, adhesive texture, and aromatic smell when scratched by a fine scalpel. The nature of phases 2 and 3 and whether they are indeed separate constituents was first examined through nanoindentation (Figure 2c,d). A linear path was assigned, comprising 13 probing locations along the plane of the longitudinal and latitudinal sections (Figure 2c). At each location, a 0.1 × 0.1 mm square area was selected and divided into a 10 × 10 grid of data points, which were then indented. The force‐depth (*P*−*δ*) curves were recorded at each point by applying displacement‐controlled indentation. The sample was indented until reaching the peak force, *P*_max_, after which the indenter returned to its initial position.

This withdrawal process registered a finite force generated by elastic recovery, which, in turn, was used to predict the local elastic moduli (*E*) at different locations (Figure 2d). Equations 1 and 2 were used to predict the local elastic moduli (*E*). Figure 2e depicts the location of the data points, where the color indicates the respective magnitude of *E* for the longitudinal and latitudinal sections. The *k*‐means image segmentation of the three different phases is shown below each indentation data point. By comparing the location and the magnitude of *E* with its location on the segmented images, each data point was associated with its corresponding phase and assigned a similar color (Figure 2e). The values appear to cluster into three bands with average elastic moduli of 0.86 kPa, 6.9 kPa, and 4.0 kPa for phases 1, 2, and 3, respectively. The measurements suggest that phases 2 and 3 are either distinct constituents or possibly fibers aligned in two different directions, leading to different elastic moduli.

### 2.3 Microscopic and Spectroscopic Characterization of the Fibers

**FIGURE 3:**
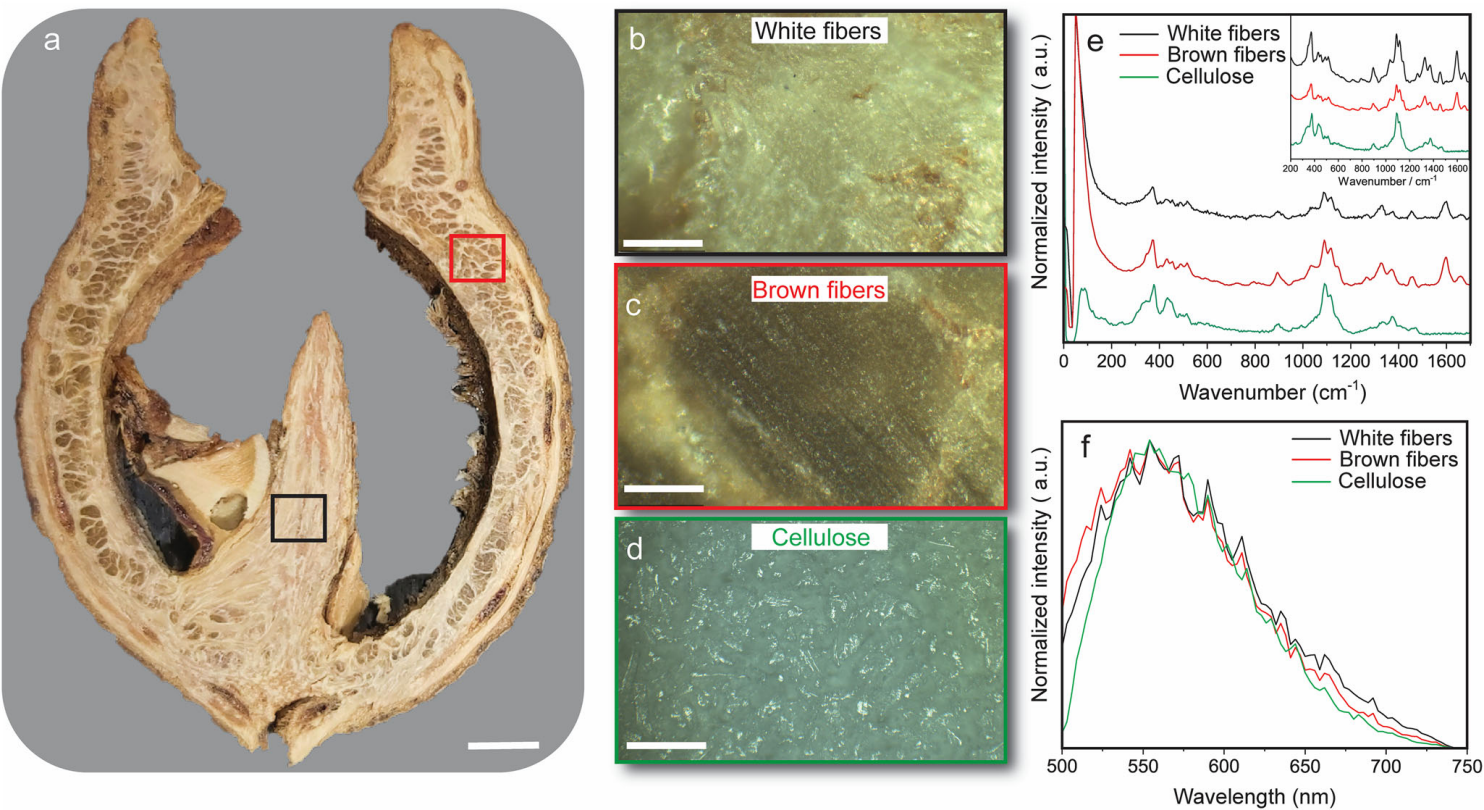
Optical micrographs, and Raman and fluorescence spectra of different regions of the nut. (a–d) Optical images of (a) an entire transverse section of a nut, (b) white fibers, (c) brown fibers, and (d) pure cellulose. (e) Raman spectra recorded at different regions of the nut, representing the chemical composition of each phase (brown fibers, white fibers, and pure cellulose). (f) Fluorescence emission spectra acquired on the brown fibers, white fibers, and pure cellulose. The scale bar in panel a is 3 mm, and the scale bars in panels b–d are 100 µm.

Plant fibers have garnered considerable attention for their applications as reinforcements in natural fiber composites [4, 15]. Their mechanical properties may be linked to the levels of secondary metabolites, hemicellulose, pectin, and polyphenolic substances (lignin) [16]. From the different gradations of color intensity in the CT scans in Figure 1d,e, it is inferred that the observed two phases likely represent fibers with different orientations or densities. The optical depiction of the corresponding phases, serving as sources for the Raman spectra, is illustrated in Figure 3a–d. Figure 3b represents phase 2, characterized as white fibers, and Figure 3c represents phase 3, presented as brown fibers. The Raman spectra obtained from phases 2 and 3 are similar, indicating high similarity in chemical composition (Figure 3e), and the characteristic cellulose peaks in these spectra (373, 1095 cm^−1^) [17, 18, 19] reveal their cellulose‐rich nature. Additional peaks were also observed at 1596 and 1656 cm^−1^ in both the brown and white fibers but not the pure cellulose, suggesting the presence of lignin/lignocellulose components. This finding was also corroborated by the fluorescence characteristics of the two phases. It is important to acknowledge the existence of diverse cellulose phases within the spectral range 550–600 nm (Figure 3f) that stem from the possibility of glycosidic bonds between glucose molecules assuming a double bond configuration [19]. This analysis suggests that, while the chemical composition of the phases remains consistent, disparities in mechanical properties are likely attributed to variations in fiber density and orientation.

While the mechanical properties of the marri nut can be related to its chemical makeup, we anticipated that the extraordinary robustness of the overall structure is linked to its nearly spherical shape and composite construction, where the soft matrix provides an energy‐dampening matrix through which fiber bundles are intertwined and oriented in various directions. This is analogous to synthetic composite materials, where fibrous components are known to be an essential element for reinforcement, and are therefore embedded within the structure at different orientations, for example, 30, 45, or 90°, in anticipation of several loading directions when in service [20, 21, 22]. In addition, the volume fraction of the fiber (theory of mixture) [23], and its individual makeup are crucial parameters to consider when designing an ideal composite material.

**FIGURE 4:**
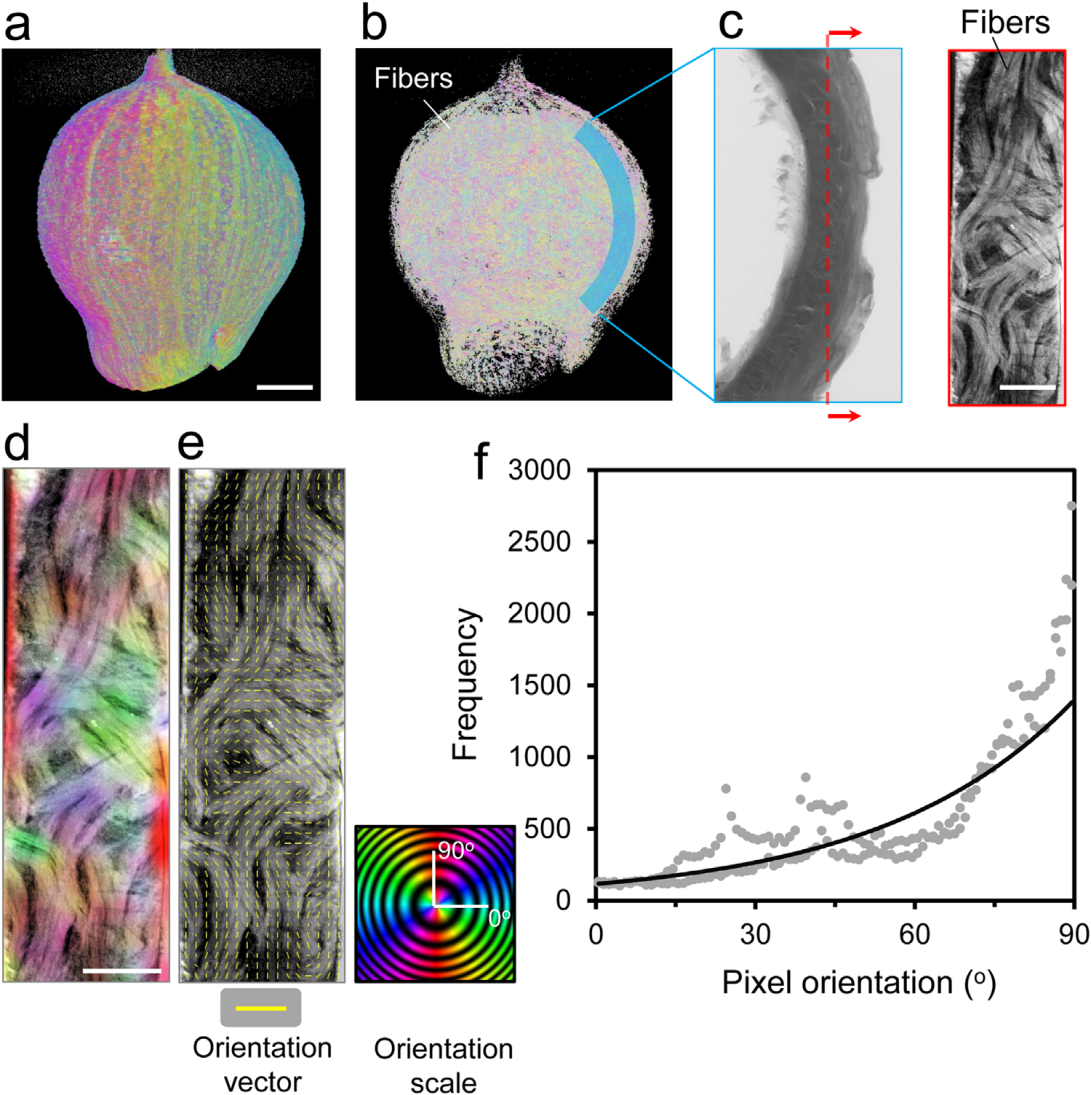
Orientation vector analysis of the fiber alignment. (a−d) CT images of the (a) nut, (b) fibers, (c) representative cross‐section, and (d) color‐coded orientation tracking. (e) Orientation vectors. (f) Distribution of the number of fibers (“frequency”) with the angle. The scale bar in panel a is 10 mm, and the scale bars in panels c and d are 3 mm.

In light of the above consideration of synthetic composites, the role of the fibrous components of the nut was examined on a representative cross‐section (Figure 4a–c) [24]. The fiber bundles reveal a diverse range of angles. This high variability and randomness in fiber orientation endow the nut with the ability to sustain loads from random directions. Figure 4c shows a magnified view of the circumferential section of the nut, revealing a high density of rough fiber bundles entangled and oriented in various directions. Directional analysis by contour maps and a vector field analysis based on the gradient structure tensor revealed that the fibers are oriented over a range of angles spanning 0−90° with respect to the horizontal axis, *x* (Figure 4d,e). Quantitatively, the vector field frequencies and their respective orientations reveal a flat and approximately exponential trend (Figure 4f). The average angle of the vector field is 54°, with a median of 58°. Almost 78% of the vector data points are >45°, and around 30% of the vector field values are in the near‐vertical direction (80−90°). This noticeable tendency toward the vertical direction originates from the fact that the nut is formed from fibers growing from the stem, through which the fibers converge, giving it its bell‐like shape. This is much more evident at the surface of the nut, where visual and tactile inspection suggests that fibers are mostly oriented toward the stem. Deeper through the thickness of the nut, however, the fibers become more random (Figure 1d,e). Cellulose, being a highly fibrous material, may also explain why these fibers are highly entangled. In the realm of textile science and clothing technology, it is known that adhesion, and thus the strength of yarns, increases with polarized fibers [25, 26]. Additionally, due to the rough and varied thicknesses of the fibers, they are potential candidates for exhibiting competitive energy absorption capabilities through inter‐fiber friction when subjected to loads, impacts, and sudden mechanical shocks.

### 2.4 Apparent Macroscopic Properties

**FIGURE 5:**
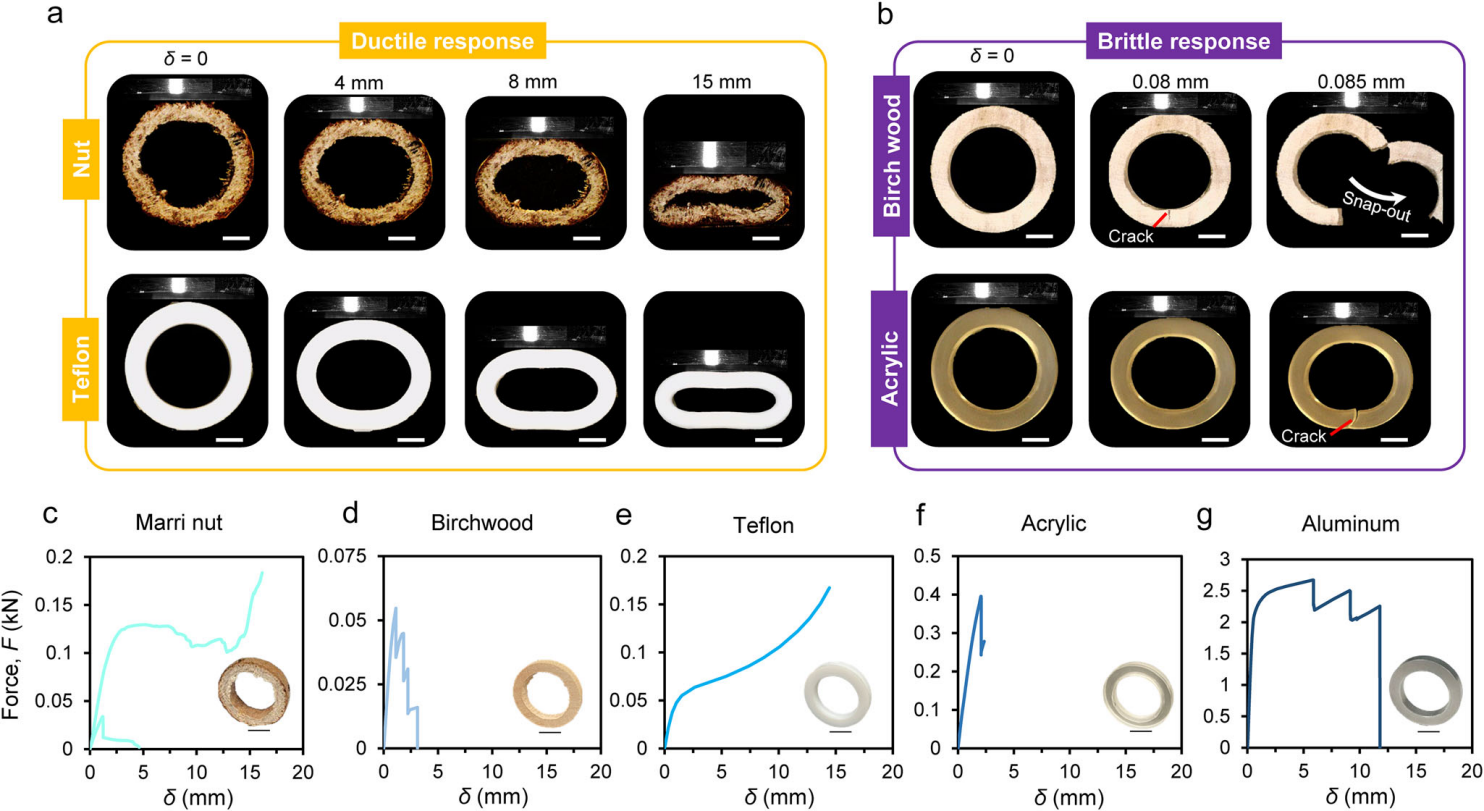
Mechanical properties of the marri nut in comparison to other commercial materials. (a) Ductile response of the marri nut and its similarity with Teflon. (b) The brittle response of birchwood and its similarity with acrylic. (c−g) Compression force‐deflection curve (*F*−*δ*). All sample rings were of identical geometry, size, and dimensions. The scale bars are 5 mm.

To assess the mechanical properties of the marri nut, ring samples (thickness, *t* = 5 mm, inner radius, *R*_i_ = 30 mm, and outer radius of, *R*_o_ = 35 mm; Figure 5) were cut out from the nut as well as several reference materials (wood, Teflon, aluminum, acrylic) and subjected to quasi‐static displacement‐controlled compression (Methods). Ring specimens were selected because extracting dog‐bone or beam samples required excessive machining that disrupts the nut's natural fiber flow. Such machining cuts along multiple orientations and results in severe damage that compromises the properties and leads to loss of representativeness of the nut's true microstructure. Ring samples, however, can be obtained with minimal disruption to fibers, preserving the natural grain flow and providing a sample that is structurally and microstructurally representative of the material. Several earlier studies adopted similar half‐ring or full‐ring sample analyses [27]. It is also worth noting that variations in the nut's moisture content can influence its mechanical properties, a parameter often controlled in previous studies through air [8] or oven drying [27, 28, 29]. In this work, all samples were placed in a desiccator for a minimum of 24 h prior to testing to ensure consistent moisture removal while preserving the natural structure of the nut. The marri nut exhibits progressive and gradual deformation, closely resembling the well‐known compliant and ductile behavior of Teflon (Figure 5a). The ductile behavior of the latter is attributed to the unwrapping of and intermolecular friction between its polymeric chains [23]. By analogy, there must be a comparable mechanism at the macro‐, meso‐, and/or micro‐scale that enables such a response from the marri nut. Anticipated deformation, pulling, and unwrapping of the fibers, coupled with simultaneous deformation of its soft phase, impart viscoelasticity, while contact friction and damage collectively contribute to its ductile behavior. In contrast, another member of the nut family, birchwood, displayed limited deformability and pronounced brittleness (Figure 5b). The accumulation of elastic energy in birchwood resulted in a sudden fracture, causing half of the ring to pop out of the structure. Indeed, birchwood contains fibers like most hardwoods to which it belongs, however, its fibers are mostly unidirectional and have organized growth rings, where initiating and thus progressing cracks tend simply to propagate along or through them with less tortuosity than is anticipated to be encountered in marri nut (section 2.5 sheds more light on this [28, 29]). Hence, the sudden fracture of birchwood. Similarly, stiff acrylic rings exhibited a brittle fracture, with cracks initiating at locations of maximum tensile stress (at the bottom inner surface of the ring), followed immediately by an unstable crack propagation, which fractured the ring (Figure 5b).

The comparison of the marri nut with other synthetic and natural materials can be further emphasized by examining the compressive force‐deflection curves (*F*−*δ*) from the compression tests. Figure 5c–g shows the *F*−*δ* curves for the marri nut, birchwood, Teflon, acrylic, and aluminum. Two ring samples were extracted from the marri nut, one each along the latitude and longitudinal sections (Figure 2a). For the nut, the compression test along the latitudinal direction exhibited a sharp linear increase in force, with stiffness of 53.2 N m^−1^, followed by softening (reduced stiffness) and an extended plateau until 15 mm of deformation was reached (75% of the sample diameter), whereupon the response rapidly hardened, marking the point of densification (Figure 4c). On the other hand, the longitudinal sample, mainly due to the presence of the nut stem, displayed the same initial linear rise in force with a similar stiffness magnitude, peaking at around 1.3 kN, immediately followed by a sudden fracture. Fractures consistently emerged around the stem due to the presence of sharp edges and abrupt changes in the geometry, likely resulting in stress concentrations. Birchwood showed a very comparable response with a small difference, having multiple fractures. The first fracture occurred at the bottom part of the ring, followed by another fracture at the inner surface of the ring (Figure 5b). The Teflon sample displayed a typical response of a polymeric structure, characterized by a linear rise, softening, and brief hardening beyond 50% of the ring diameter. In contrast, the acrylic ring exhibited a brittle response, with force rising linearly, peaking, and suddenly dropping at the onset of fracture; unlike birchwood, a single fracture was sufficient to cause a full collapse of the ring. The aluminum ring sample displayed a linear rise, followed by softening and multiple stable crack propagations due to the inherent ductility of aluminum, until the complete plastic collapse of the sample.

### 2.5 Damage and Fracture Resistance

**FIGURE 6:**
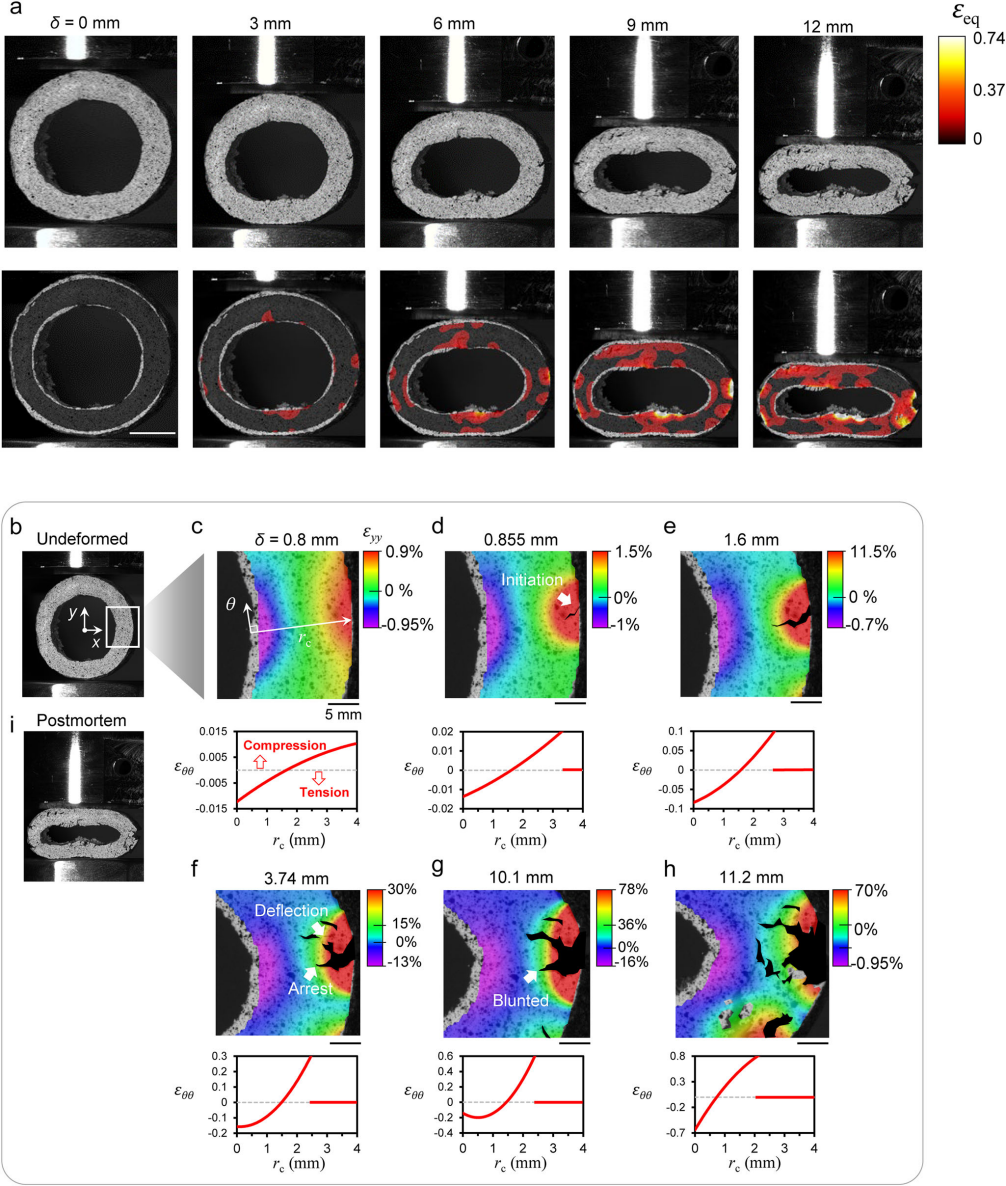
Damage assessment and crack(s) extension within the nut. (a) Progressive increase in equivalent strain (von Mises strain) with increased deflection: *δ* = 0−12 mm. (b) Undeformed, pristine ring sample. (c−h) equivalent strain contour plot with increased deflection, *δ* = 0.8, 0.855, 1.6, 3.74, 10.1, and 11.2 mm, around a macrocrack. Under each contour plot, we show the corresponding angular strain (*ε_θθ_*) profile at the radial axis (*r*_c_) that coincides with the crack plane. (i) A fully compressed sample. The scale bar is 5 mm.

We have thus far noted two key attributes of the marri nut: (*i*) it is composed of a material made up of two phases and a highly intertwined network of fibers, and (*ii*) the nut takes the shape of a spherical‐bell shape, forming nearly perfect circular cross‐sections. In light of these assets, a qualitative analysis of the fracture resistance was conducted, particularly because it has demonstrated significant ductility and an impressive ability to withstand substantial strains without losing its load‐bearing capacity. To this end, the lateral section (Figure 2a) of the nut was examined under quasistatic compression while monitoring its deformation in situ with digital image correlation (DIC). This provides the displacement field and the corresponding strain field (von Mises strain), *ε*_eq_, for different values of the deflections of the loaded section, namely *δ* = 0, 3, 6, 9, and 12 mm (Figure 6a). Large values of *ε*_eq_ correspond to regions where cracks typically initiate and progress during compression. As shown in Figure 6a for *δ* > 3 mm, cracks are initiated at the bottom/top inner surface and left/right outer surface of the ring sample, where the highest tensile stresses are measured. As *δ* increases, the nut accumulates more strain, and the outer surface of the sample clearly exhibits signs of significant damage with visible cracks and strains exceeding *ε*_eq_ > 0.6. The damage is not localized but rather distributed over the entire nut. The nut ring does not fracture into separate pieces, despite the maximum equivalent strain reaching a value of around 74%. A closer examination of one of the major macrocracks that was initiated on the outer right surface of the ring reveals an incremental propagation of the crack as *δ* increases (Figure 6c–h) due to the densely packed fibers combined with the viscoelastic phase (the soft matrix), which slows down the crack propagation. Multiple crack deflections and branching, along with the irreversibility of viscoelastic deformation, help absorb and dissipate elastic energy, thereby reducing the strain energy release rate that suppresses crack propagation [30, 31].

To track the strain along the crack, the entire strain tensor was rotated to a local cylindrical frame (*θ*‒*r*_c_) using a rotation matrix (see Methods), where *r*_c_ is measured along the crack plane's axis (Figure 6c). The strain was calculated normal to the crack plane, i.e., angular strain, 𝜀*_θθ_*, which directly results from the concomitant normal stress component normal to the crack plane. Under each of the contour plots, 𝜀*_θθ_* is plotted along the radial axis *r*_c_ in Figure 6c–h. Analogous to curved beam theory, we first observe in all plots that there is a circumferential plane (along the angular direction) at which the strain switches direction from compression for a small value of *r*_c_ to tension. This results from the near‐perfect circular cross‐section of the nut, which ensures that its inner part remains under compression while its outer part is under tension. We also observe that the tensile portion of the ring sample is most susceptible to crack initiation and propagation (Figure 6c), and that at points coinciding with crack initiation and extension, the angular strain vanishes (Figure 6d–h).

Figure 6d,e shows that, despite the crack extending from 1.4 to 1.5 mm (*δ* = 0.855 to 1.6 mm) through the nutshell, the *ε_θθ_*−*r*_c_ profile still shows compressive strains beyond *r*_c_ < 1.5 mm. Consequently, as the crack approaches *r*_c_ = 1.5 mm, it slows down and is eventually arrested near *r*_c_ ≈ 2.45 mm (1.6 mm into the shell's thickness; Figure 6f). The buildup of strain energy from compression manifests itself as cracks branching and deflecting, which is a clear sign of energy dissipation within the structure [20, 22]. The deflected cracks were not able to propagate beyond the circumferential plane of *r*_c_ = 2 mm because they required tensile stresses and normal strains to feed their propagation [31]. It can also be observed that at *δ* = 10.1 mm, the main macrocrack gets blunted, causing its root radius to increase, which reduces the stress intensity factor of the crack and its likelihood to extend further (Figure 6g). Given that multiple studies on fibrous materials have reported similar findings, we confidently attribute this crack blunting to the presence of the fiber network [32]. Finally, at *δ* = 11.2 mm, the deflected cracks further branch into smaller cracks, spreading across the entire nut. This crack branching has the advantage of distributing stresses around stress concentration points [21]. Indications of crack bridging [33] can be noticed, as several isolated cracks can be seen dispersed in the vicinity of the macrocrack (Figure 6h). There may be, however, other additional toughening mechanisms, like fiber bridging behind the macrocrack or microcrack coalescence, that our analysis has not yet revealed.

As shown in Figure 6i, the initiation of cracks in the nut does not compromise its integrity, which is typical of composites with fiber networks [32, 34, 35]. Such networks, in general, can dissipate the applied energy through fiber reorientation, fiber fracture, bond breaking, and fiber slippage (friction) [36]. Effective crack deflection along and through the fiber depends on the relative elastic moduli of the fiber and the soft matrix [37, 38]. The nanoindentation experiment (Figure 2e) revealed an elastic modulus ratio of approximately 6.3 between the fiber and the soft matrix, which may hold significance in designing similar composites inspired by the microstructure of the nut. The interaction between the soft matrix and the fibers leads to progressive and stable crack propagation, combined with multiple crack deflections, resulting in distributed damage and crack bridging. These factors enable the nut to undergo significant deformation and dissipate the applied input energy, ultimately providing maximum protection to the seeds within.

### 2.6 Biomimicry of the Marri Nut

**FIGURE 7:**
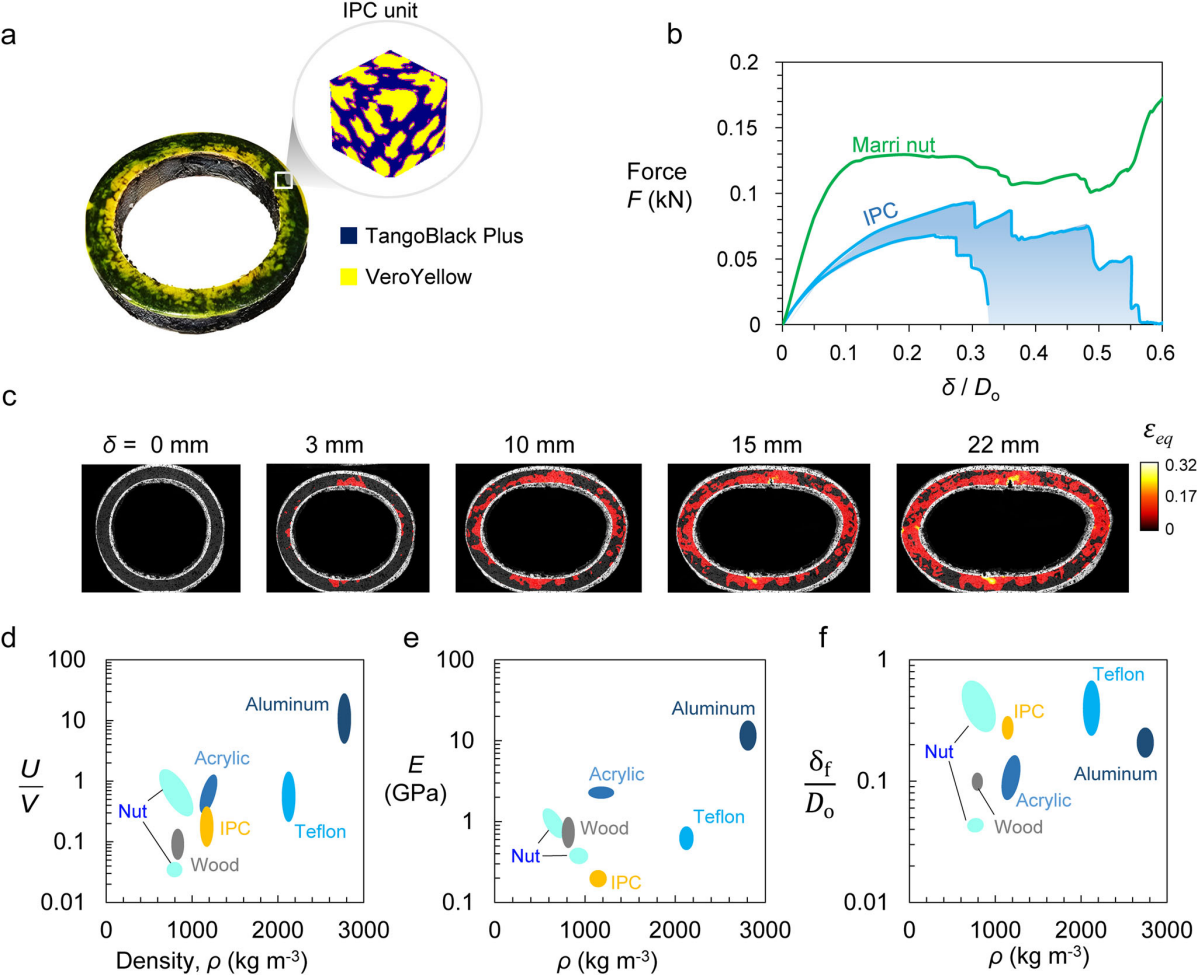
A bioinspired design from the marri nut. (a) A bioinspired ring sample made out of an interpenetrating phase composite unit cell (shown in the inset). The soft material (TangoBlack Plus) is in blue, and the hard material (VeroYellow) is in yellow. (b) Force‐deflection curve of the IPC along with the ring sample for comparison. (c) Equivalent strain contour plot with increasing deflection, *δ* = 0.8, 3, 10, 15, and 22 mm. (d–f) Ashby charts of absorbed energy *U* per volume *V* (i.e., *U*/*V*), Young's modulus *E*, and normalized maximum deflection, *δ*_f_ / *D*_o_, with corresponding material density *ρ* for the marri nut and some common materials. All sample rings were of identical geometry, size, and dimensions, except for the IPC sample, necessitating the normalization of all metrics.

Among the many attributes of the marri nut, one notable aspect we aim to reproduce was its microstructure, which is composed of highly intertwined fibers and multiphase interpenetrating composites made of stiff fibers and soft matrix material. Specifically, we sought to redesign and fabricate a ring sample using composite materials, forming an interpenetrating phase composite (IPC) of two mechanically contrasting materials: a soft material, namely TangoBlack Plus, mirroring the soft matrix, and a hard material, VeroYellow, that represents an analogy to the stiff fibers in the natural marri nut. These two materials are reliable and compatible options used in PolyJet 3D printing technology. A 3D‐printed sample with a 50% volume fraction of VeroYellow (in yellow) to TangoBlack Plus (in blue) is shown in Figure 7a, while the inset represents the unit block sample from the IPC used in the design and fabrication of this bioinspired ring sample. The distribution of the two phases is shown in Figure S4, capturing the multilayered and interpenetrating arrangement that follows the overall curved configuration of the nut. In this design, we reproduced the general shape of the nut, the elastic modulus contrast between the two phases, and their positional distribution during 3D printing. However, the model does not capture fiber pull‐out or inter‐fiber entanglement through contact. Because these fiber‐level interactions are key contributors to both intrinsic and extrinsic crack‐toughening mechanisms, the 3D‐printed sample may not fully reflect these important features. Future work will need to incorporate actual fiber elements into the composite to better emulate the fibrous interactions of the natural nut. Overall, while our approach does not fully reproduce the biological hierarchy and fiber mechanics, it successfully captures the essential topology‐driven deformation and toughening behavior.

Figure 7b shows the force‐deflection (*F*−*δ*) curve in blue for the ring sample subjected to displacement‐controlled compression as previously mentioned (Methods). Interestingly, the mix of these two contrasting materials, fused together in a randomly interpenetrating manner, allowed the IPC to exhibit progressive and relatively high deformability similar to the nut sample (also shown in green on the same plot). We have tested several samples, and most responses lie within the shaded region shown in Figure 7b. Despite VeroYellow being a relatively stiff and brittle material, when mixed with the soft TangoBlack Plus, it creates a composite that collectively provides a ductile response, allowing the part to exhibit multiple fractures while maintaining load‐bearing capacity and dissipating substantial energy (represented by the area under the curve). These incremental damages appear as successive drops in force, reflecting crack nucleation and/or extension. This behavior contrasts with the wood sample shown earlier in Figure 5b and the acrylic sample. We may thus infer that the IPC strategy, mimicking the two interpenetrating phases of the marri nut, enables damage resistance in the synthetic IPC counterpart.

Figure 7c shows the progressive compression of the IPC sample under in situ monitoring of the DIC system (as described previously; Methods). We report here the relative dispersion of damage across the sample via the equivalent strain field contours. The IPC managed to distribute the local deformation of the material, thereby engaging the entire body of the sample in terms of damage, similar to the natural nut sample. The notable difference is that the amount of local strain in the IPC sample is collectively 50% less than that of the natural nut, which underwent strain up to 0.74. Of course, other aspects of natural fibers are not yet captured in this version of the bioinspired IPC sample; aspects such as the highly intertwined fibers across the body of the material and the potential for untangling and sliding (thus friction dissipation) upon loading are still absent in the IPC design.

Using the force‐deflection curves in Figures 5c–g and 7b, we calculated the corresponding energy absorption (area under the *F*−*δ* curve), the effective Young's modulus *E*, and the deformability, which is the normalized maximum deflection *δ_f_*/*D*. For the marri nut, the radius‐to‐thickness ratio, *R*/*t* = 2.64, does not meet the thin curved beam criterion (*R*/*t* > 10). Therefore, Roark's thick curved beam equations were used to estimate *E* of the ring (Equation 7, Methods) [39]. Ashby‐like bubble plots for the energy absorbed per volume, *U*/*V*, the apparent elastic modulus, *E*, and the final normalized deflection, *δ_f_*/*D*, against the density of the material, *ρ*, are shown in Figure 7d–f. The IPC sample shows an average performance when compared with other synthetic materials, having comparable toughness (*U*/*V*) to that of Teflon and superior to wood. In terms of elastic modulus, *E*, the IPC sample is inferior to the rest, which is attributed to the soft TangoBlack Plus constituents (∼50% of its volume) within the IPC. However, as depicted in the DIC analysis of Figure 7c, the IPC exhibits competitive deformability that is comparable to Teflon and the nut sample, surpassing the remaining materials shown in the chart.

In addition to the IPC‐based prototype, we conducted a preliminary study to examine the effect of conventional continuous fiber reinforcement on the mechanical response of the bioinspired ring structure. A ring specimen incorporating approximately 10% continuous carbon fibers was fabricated using a commercially available composite 3D printing system and tested under the same displacement‐controlled compression protocol as the non‐reinforced structure. As shown in Figure S6, the incorporation of continuous fibers leads to a pronounced enhancement in mechanical performance, with the fiber‐reinforced sample reaching a peak force of approximately 0.50 kN at a displacement of ∼3.5 mm, compared with ∼0.15 kN at 4 mm displacement for the non‐reinforced structure, corresponding to an approximately three‐fold increase in load‐bearing capacity. This result demonstrates that even a relatively small volume fraction of continuous fibers can substantially enhance the mechanical response of the bioinspired design, supporting the role of fibrous reinforcement in improving load transfer and toughness, consistent with the stress‐dissipation mechanisms identified in the natural marri nut.

Overall, the marri nut still stands out due to its light weight, offering competitive mechanical properties at a relatively lower density, and it consistently occupies the mid‐top left corner of the materials property plots. For instance, the marri nut exhibits an energy absorption density of 1.15 J cm^−3^, which is 1.3, 15.7, 1.5, and 0.05 times that of acrylic, birchwood, Teflon, and aluminum, respectively, while being lighter than all of them. In terms of *E*, the marri nut outperforms birchwood but falls short of the rest. Remarkably, as pointed out above, the nut shows highly ductile behavior, with a normalized final deflection, *δ*_f_/*D*, surpassing that of the other materials. Clearly, the marri nut possesses powerful toughening mechanisms, enabling it to undergo extensive deformation while maintaining a high load‐bearing capacity.

## 3 Conclusions

The outcome of this study revealed the following key features of the marri nut: The nut takes a bell shape, with its shell made out of hard, relatively dry 5 mm shell. Deeper into the shell, it becomes more fibrous, mixed with an aromatic soft matrix. 3D and 2D imaging slices, along with machine learning segmentation accompanied by indentation, revealed three phases of materials. Raman and fluorescence spectroscopy revealed that phases 2 and 3 are mostly cellulose with varied densities and levels of lignin/lignocellulose derivatives, while phase 1 is resin. The nut is composed of fibers woven within a soft matrix, where fibers are oriented in all directions, but 78% of the vector field mapping the orientation of the fibers is >45°, toward the vertical direction, that is, toward the stem of the nut, from where the fiber grew. The interplay of fiber breakage, pullout friction of fibers, and viscoelasticity of the matrix enables the nut to exhibit remarkable damage resistance and “ductility” that is on par with plastics like Teflon. The nut stands out in terms of mechanical energy absorption and deformability when compared with birchwood, Teflon, acrylic, and aluminum. The nut is also fracture‐resistant; macrocracks within the nut fail to extend beyond the mid‐section of the nut shell. The cracks move slowly because of the viscoelasticity of the soft matrix and the dense fibers. We have also seen evidence of multiple crack deflections along the fibers, accompanied by fiber pullout and crack blunting within the nut. These characteristics of the marri nut make it highly deformable, possess high‐energy absorption density structure on par with Teflon, and have a comparable elastic modulus to that of acrylic, all while being exceptionally lightweight. This positions the marri nut as a significant model for advanced material science in the development of protective gear and similar applications. Our study focuses on examining fiber architecture and its relation to the macroscopic mechanical properties of the nut; to gain further insights into nanoscale structures, it would be valuable to employ advanced techniques such as small‐angle X‐ray scattering (SAXS) in future studies. Inspired by the unveiled origins of toughness of the marri nut, a composite structure was engineered using two contrasting materials that mimic the natural nut's soft resin and stiff fibers. Mechanical testing of the engineered material demonstrated high toughness, highlighting its potential as a bioinspired material for the design of ultra‐tough armor, protective devices, sportswear, tires, greenhouse covers, and medical gloves. It is worth noting that the current prototype is the closest possible approximation of the microstructural complexity of the nut, as no additive manufacturing method can yet produce multilayer structures with fiber bundling and feature sizes at this scale. By incorporating features such as highly intertwined fibers and their sliding interactions or fiber pull‐out effects into the composite structure, we envision that its toughness and elastic modulus could be further enhanced.

## 4 Experimental Section

### 4.1 Computed Tomography (CT)

CT scans were acquired using the X VIEW CT X5000 scanner (North Star Imaging), employing a working voltage of 100 kV and 150 µA. The nuts were securely positioned and subjected to scanning at a speed of 7.5 frames per second. Furthermore, an X‐ray micro‐CT system, SkyScan 1272 CMOS (Bruker), with a working voltage of 50 kV and 200 µA, scanning at a rate of 3 frames per second, was used on smaller segments.

### 4.2 Samples

Nuts from the marri tree (*Corymbia calophylla*) were harvested in January and February of 2020 in Western Australia. All samples were kept in a humidity‐controlled chamber for at least 24 h before any mechanical testing to ensure constant moisture content. The dimensions of the nuts were measured using a Vernier caliper with an accuracy of 0.1 mm.

### 4.3 Image Segmentation with k‐Means

High‐resolution pixelated images of 600 dpi were fed into Matlab [40]. Accordingly, we employed the k‐means clustering algorithm to segment the images into different distinct segments [41]. The number of segments or clusters is a user input to the algorithm; therefore, we conducted a sensitivity analysis by increasing the number of expected clusters from 2 to 10. However, beyond 4 clusters, the overall distribution of clusters remained unchanged, but it displayed finer subdivisions of the major partitions. Consequently, we limited the segmentation to four regions, including the background.

### 4.4 Additive Manufacturing

The commercial CAD software Materialise Magics was used to construct the interpenetrating phases by performing Boolean operations after acquiring the files from the CT scans. The samples were then fabricated using a J750 resin‐based 3D printer (Stratasys). For this analysis, we selected VeroYellow as the reinforcement phase (a rigid, opaque photopolymer) and TangoBlack Plus as the matrix (a soft, rubbery material forming the interpenetrating phase). For continuous carbon fiber reinforcement, ring specimens were fabricated using an Onyx Pro system (Markforged) with an Onyx matrix and approximately 10% continuous carbon fibers.

### 4.5 Nanoindentation

An Agilent G200 nanoindenter equipped with an XP head and a Berkovich diamond indenter was used to acquire the load‐displacement curves, where a total of 10 indents were made at a depth of 700 nm. The samples were prepared by sawing a relatively flat sample of the outer shell and using a crystal bond to mount it. The location of interest was indented to a maximum depth, *δ*_max_, with an indentation force, *P*_max_. The direction of indentation was then reversed to withdraw the indenter back to its initial position. Shortly after the withdrawal of the indenter, there was a brief elastic recovery by the material to regain its original shape, but this was prevented by permanent plastic deformation, leaving a permanent impression on the surface. This brief elastic recovery was used to estimate the local elastic modulus, *E*, of the indented material by computing the slope just after reversing the indentation direction, as per the Oliver‐Pharr method [42] (Figure 2c–e). The slope d*P*/d*δ* was computed at the instant of reversing the direction of indentation, which captures the localized, brief elastic recovery of the material. The elastic modulus was calculated using the Oliver–Pharr method:

(1) $$E=\frac{1}{2}\frac{dP}{d\delta }\frac{\sqrt{\pi }}{\sqrt{A}}$$

where *A* is the area of contact, and the Berkovich indenter is given by:

(2) $$A=24.5\;\delta_{c}^{2}$$

where $\delta_{c}=\delta_{\max}-0.75\frac{P_{\max}}{dP/d\delta }$.

### 4.6 Quasistatic Compression and Computation of Young's Modulus of Ring Sample

Steel plates connected to a universal testing machine (Instron 5960 series) compressed the ring samples at a displacement rate of 0.1 mm s^−1^. A low‐profile 5 kN load cell connected to the steel plates measures the compressive force during the compression of each ring until complete crushing of the ring sample is achieved. To calculate the effective Young's modulus of thick ring samples, the approximate formula provided by Roark was used [42]. The Young's modulus of the material was estimated from the force‐deflection curve within the linear elastic regime for thick rings (*R*/*t* < 8):

(3) $$E=\frac{R^{3}}{I}\left(\frac{\pi k_{1}}{4}-\frac{2k_{2}^{2}}{\pi }\right)\left(\frac{dF}{d\delta }\right)$$

where $\frac{dF}{d\delta }$ is the slope of linear elastic part of the curve, and *I* is the moment of inertia:

(4) $$I=\frac{t^{4}}{12}$$

while the constant *k*_1_ and *k*_2_ are correction factors proposed by Roark to account for thick beam and they are:

(5) $$k_{1}=1-\alpha +\beta \;\mathrm{and}\;k_{2}=1-\alpha$$

For a rectangular cross‐section, *α* = 1.2. The offset, *e*, increases with greater thickness, which explains why it is typically disregarded for thin rings. This offset can be expressed as:

(6) $$\alpha =\frac{e}{R}=1-\frac{2}{\frac{2R}{t}\ln\left(\frac{R/t+1/2}{R/t-1/2}\right)}$$

And, $\beta =2f\left(1+\nu \right)\frac{e}{R}$, where *f* is the shape factor for different cross‐sections, that is, for a rectangular cross‐section, the shape factor is *f* = 1.2. ν is the Poisson's ratio of the material, that is ν ≈ 0.3 for wood. As such, the factor β becomes: $\beta =3.12\frac{e}{R}$. Finally, we can now estimate the apparent elastic modulus of the tested rings by calculating the stiffness of the *F*−*δ* curve in the elastic regime, specifically at *δ* = 0 in Equation 3. This approach allows us to obtain an expression for the apparent elastic modulus of the ring as a function of geometry and stiffness. Additionally, we record the final deflection, δf, at the point of fracture, which reflects the deformability of the tested materials.

### 4.7 Transformation of the Strain Tensor

To orient the strain tensor normal to the crack plane, the orientation of the crack plane was first determined by tracking the (*x*, *y*) coordinates of the crack at each frame (i.e., **E**(*x*, *y*)). These coordinates of the crack were then fitted to a linear line, from which we computed the overall slope, represented by tan *θ*. This slope was then used to calculate the orientation angle of the crack plane, denoted as theta. Subsequently, we applied a rotation matrix to the entire strain tensor at the (*x*, *y*) coordinates of the crack plane within the Cartesian coordinate system. This transformation is defined as follows:

(7) $$E\left(r_{c},\theta \right)=R\;E\left(x,y\right)\;R^{T}$$

where rotation matrix is expressed by:

(8) $$R=\left[\begin{array}{cc} \cos\theta & -\sin\theta \\ \sin\theta & \cos\theta \end{array}\right]$$

The superscript *T* denotes the transpose. Equation 7 transforms the strain tensor acting on the *x*−*y* plane to *r*_c_−*θ* direction. From this, we extracted the strain component *E*_22_ = *ε*_*θθ*_ which is the strain normal to the crack plane. This is reported in Figure 6.

### 4.8 Digital Image Correlation

A non‐contact displacement and strain measurement was conducted using the VIC 3D 9 system (Correlated Solutions). The system comprises two high‐definition, quasi‐static stereo‐imaging setups that track images of the deforming nut during testing. Speckled images were subsequently post‐processed using VIC‐3D digital image correlation software to obtain displacement and strain spatial fields at different frames of the tested nut sample.

### 4.9 Raman Spectroscopy

The Raman spectra of various phases of the nut were acquired on a Witec Alpha 300 confocal Raman spectrometer equipped with a 20× objective. A 785 nm laser was utilized, and the total acquisition time was set at 60 s with 10 accumulations.

### 4.10 Fluorescence Microscopy

The confocal fluorescence imaging and spectrum acquisition were performed on a TCS SP8 microscope (Leica) using a PicoQuant PDL 880‐B 40 MHz pulsed 405‐nm diode laser as an excitation source.

### 4.11 Statistical Analysis

All reported data points represent averages over the tested samples. No *p* values are reported, as no null hypothesis was formulated or tested in this study. Variability in the stress deflection behavior is captured in the stress deflection plots, where the shaded regions or bubbles surrounding each curve represent the spread of the experimental data across all tested samples. Each plotted data point corresponds to the mean response of the samples, while the extent of the shaded area reflects the observed scatter in the measurements. Standard deviations or other inferential statistics were not calculated, as the analysis focuses on comparative mechanical behavior and reproducibility rather than hypothesis testing.

## Funding

This material is based on works supported by Tamkeen under NYUAD RRC Grants No. CG011 and CG015.

## Conflicts of Interest

The authors declare no conflicts of interest.

## Supporting information




**Supporting File 1**: advs74669‐sup‐0001‐SuppMat.docx.

**Supporting File 2**: advs74669‐sup‐0002‐Supplementary Video 1.mp4.

**Supporting File 3**: advs74669‐sup‐0003‐Supplementary Video 2.mp4.

**Supporting File 4**: advs74669‐sup‐0004‐Supplementary Video 3.mp4.

## Data Availability

The data that support the findings of this study are available from the corresponding author upon reasonable request.
