# Supplementary material for: On the Origins of Toughness in Corymbia calophylla (Marri Tree) Nuts
Source: Adv Sci (Weinh). 2026 Mar 25;13(28):e15273. doi: 10.1002/advs.202515273 (PMC13185881; doi:10.1002/advs.202515273)
Supplement: Supplementary file 1 — Supporting File 1: advs74669‐sup‐0001‐SuppMat.docx. [file ADVS-13-e15273-s004.docx]

Supporting Information

**On the Origins of Toughness in *Corymbia calophylla* (Marri Tree) Nuts**

*Wegood M. Awad,^1^ Ahmed S. Dalaq,^2,3^ Marieh B. Al-Handawi,^1^ Rezo Getsadze,^1,4^ Oraib Al-Ketan,^1^ James Weston,^1^ Mohammed Daqaq,^5,6,7^ Panče Naumov^1,7,8,9*^*

This Document includes:

Legends for Movies S1 to S3

Figure S1-S6

Other supporting materials for this manuscript include the following:

Movies S1 to S3

Figure S1-S6

Captions to the supporting movies

**Movie S1 (separate file).** Computed Tomography (CT) video showing the cross-sectional slices at different parts of the nut along the *z*-axis. The scan starts from the seed dispersal hole at the base and ends at the stem.

**Movie S2 (separate file).** Computed Tomography (CT) video showing the lateral slices at different parts of the nut along the *x*-axis.

**Movie S3 (separate file).** Uniaxial compression of the Marri nut ring demonstrates a gradual increase in equivalent strain (von Mises strain) with increased deflection. The contour plot shows the strain build up and subsequent crack extension within the structure.

Captions to the supporting figures

**Figure S1.** **Compression testing of the marri nut under three loading orientations.** Top panels show the nut positioning between the compression plates and the corresponding cross-sectional schematic indicating the direction of applied force relative to the internal crossbar structure separating the four seed pockets. Panels a–c, e–g, and i–k present optical images of the nut before loading, during crack initiation, and after fracture for orientations 1, 2, and 3, respectively. The corresponding load–displacement curves are shown in panels d, h, and l

**Figure S2.** **Force required to initiate fracture in the marri nut compared with other commonly studied nuts.** The bar chart presents fracture forces for the three marri orientations and for red and black walnuts measured experimentally, alongside literature values for macadamia,^[1]^ almond,^[2]^ common walnut,^[3]^ cashew,^[4]^ hazelnut,^[5]^ pistachio,^[6]^ white speckled kidney bean,^[7]^ and pumpkin seed.^[8]^

**Figure S3. SEM image of the marri nut shell after mechanical sectioning.** The regions marked in red indicate damage such as delamination, fiber fraying, and layer loosening. Scale bar is 2 mm.

**Figure S4. 3D segmentation of the marri nut shell.** Phases 2 and 3 are shown in pink, the soft matrix is shown in yellow, and the thin outer layer is shown in blue. Scale bar is 3 mm.

**Figure S5.** **Raman spectra of the marri nut shell.** Spectra were recorded at ten different regions representing the brown fibers, white fibers, along with the corresponding optical images of each analyzed region. The scale bars are 100 µm.

**Figure S6.** **Compression testing of ring samples with and without continuous carbon fiber reinforcement.** Force–displacement curves obtained from displacement-controlled compression testing of bioinspired ring specimens, where the black curve corresponds to the ring containing approximately 10% continuous carbon fiber reinforcement and the red curve corresponds to the non-reinforced ring. Scale bar is 5 mm.


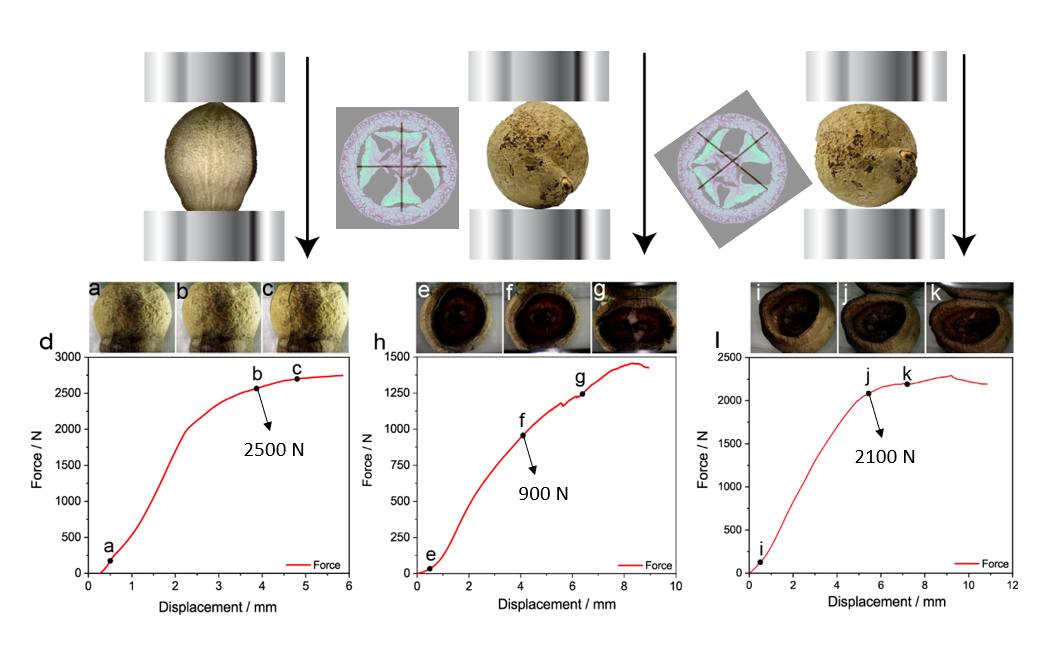


**Figure S1.** **Compression testing of the marri nut under three loading orientations.** Top panels show the nut positioning between the compression plates and the corresponding cross-sectional schematic indicating the direction of applied force relative to the internal crossbar structure separating the four seed pockets. Panels a–c, e–g, and i–k present optical images of the nut before loading, during crack initiation, and after fracture for orientations 1, 2, and 3, respectively. The corresponding load–displacement curves are shown in panels d, h, and l.


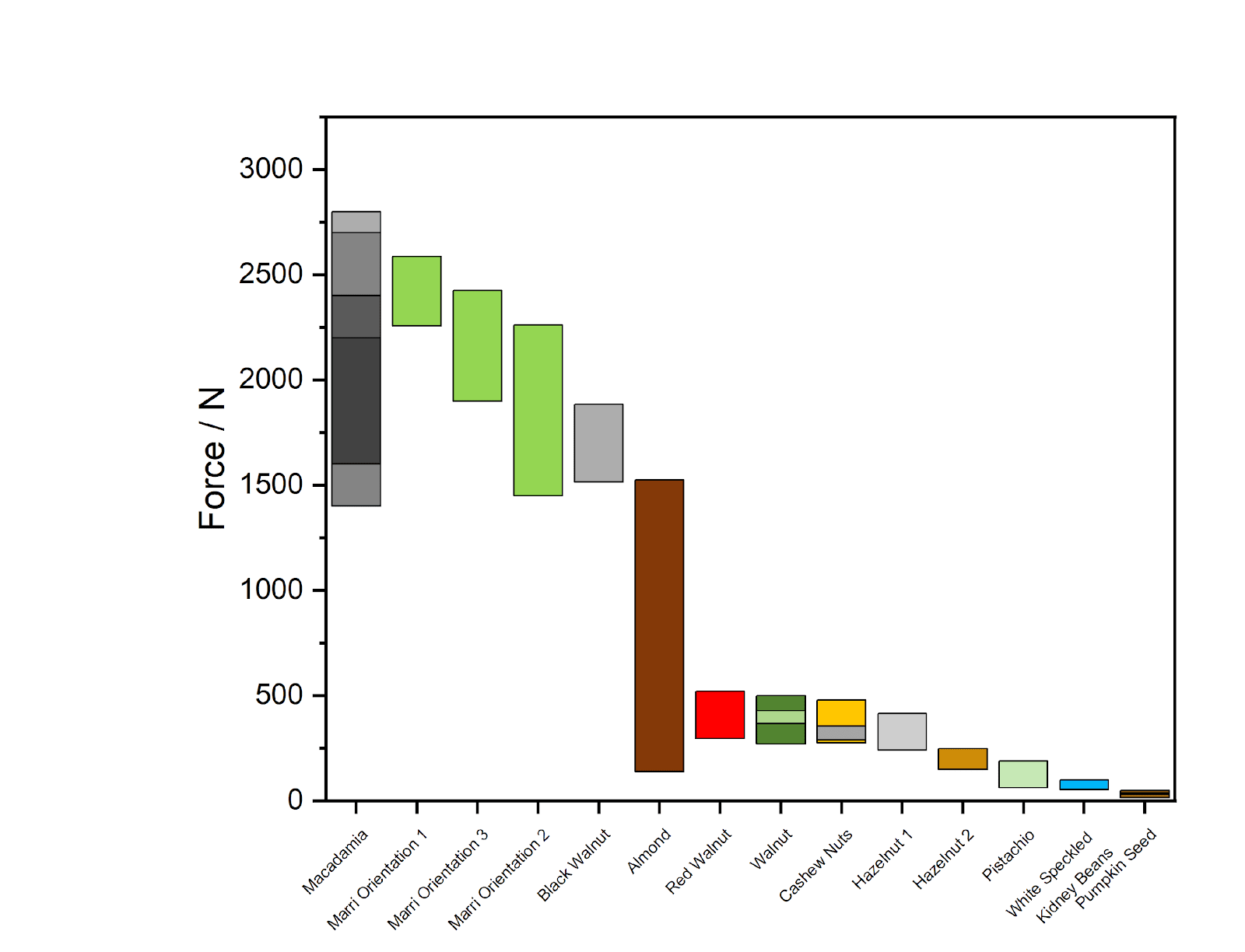


**Figure S2.** **Force required to initiate fracture in the marri nut compared with other commonly studied nuts.** The bar chart presents fracture forces for the three marri orientations and for red and black walnuts measured experimentally, alongside literature values for macadamia,^[1]^ almond,^[2]^ common walnut,^[3]^cashew,^[4]^ hazelnut,^[5]^ pistachio,^[6]^ white speckled kidney bean,^[7]^ and pumpkin seed.^[8]^


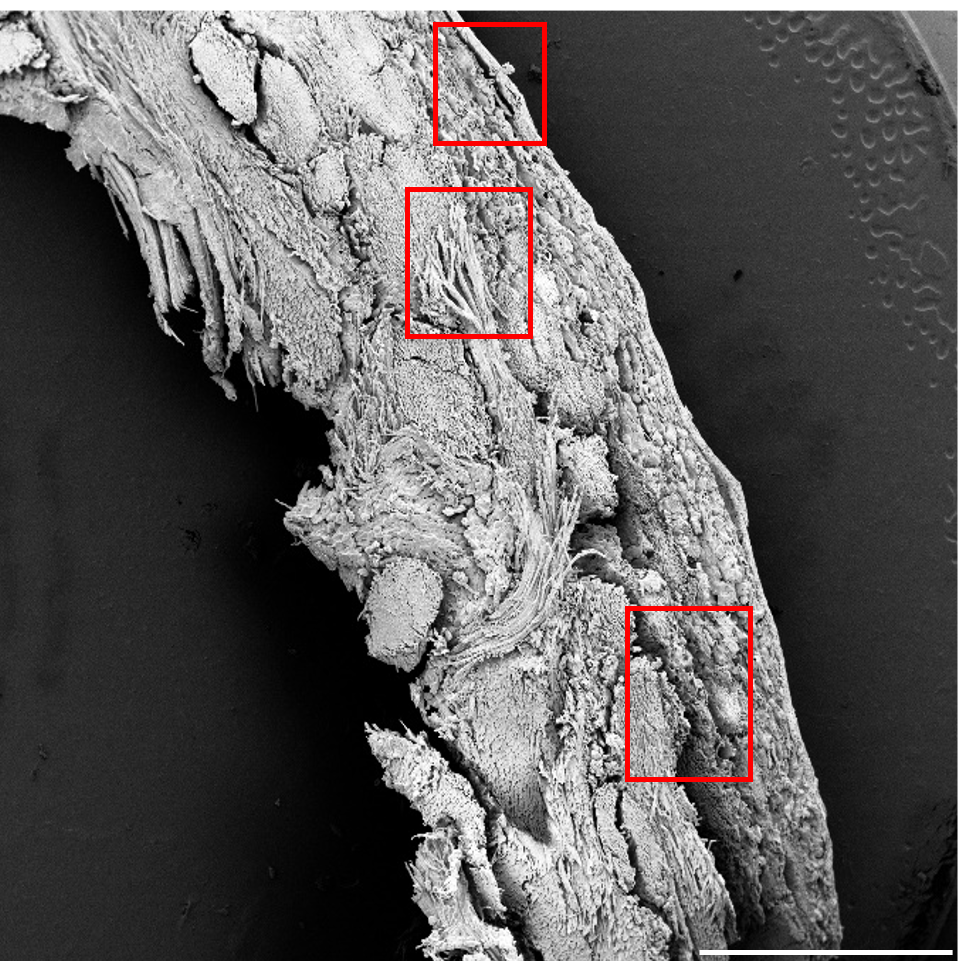


**Figure S3. SEM image of the marri nut shell after mechanical sectioning.** The regions marked in red indicate damage such as delamination, fiber fraying, and layer loosening. Scale bar is 2 mm.

**
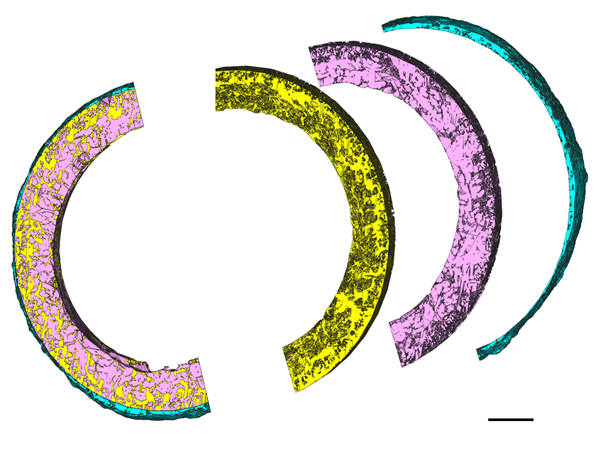
**

**Figure S4. 3D segmentation of the marri nut shell.** Phases 2 and 3 are shown in pink, the soft matrix is shown in yellow, and the thin outer layer is shown in blue. Scale bar is 3 mm


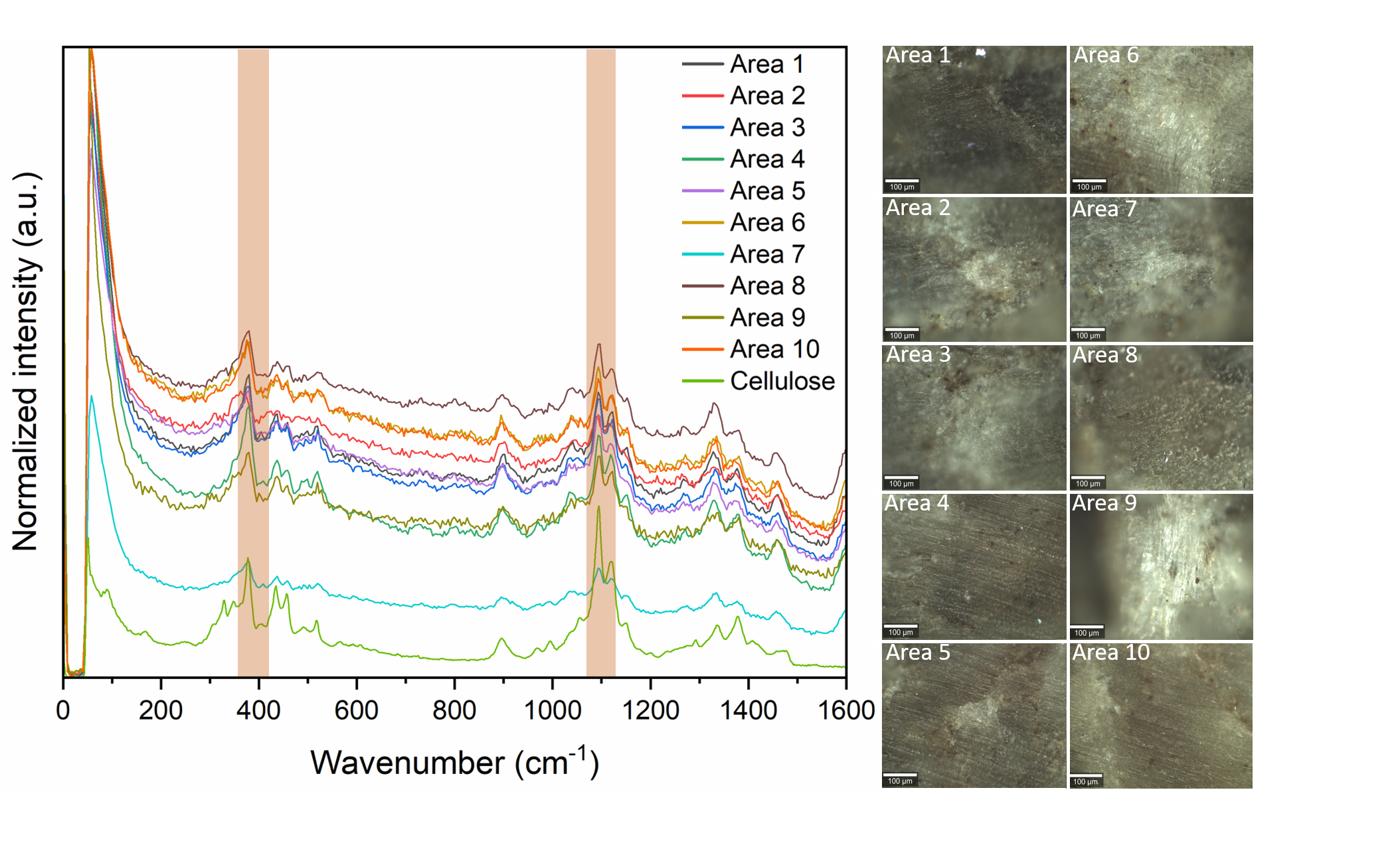


**Figure S5.** **Raman spectra of the marri nut shell.** Spectra were recorded at ten different regions representing the brown fibers, white fibers, along with the corresponding optical images of each analyzed region. The scale bars are 100 µm.


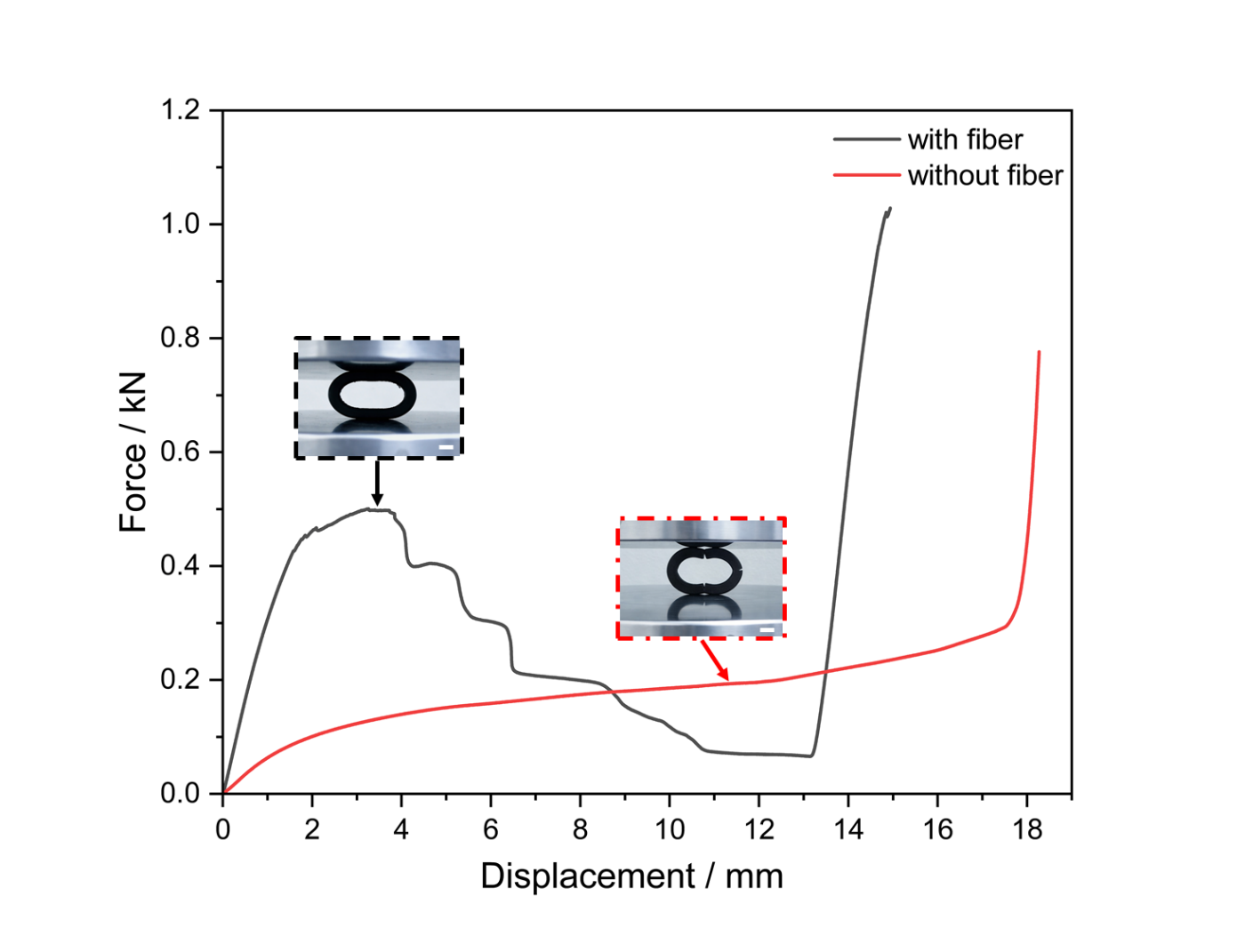


**Figure S6. Compression testing of ring samples with and without continuous carbon fiber reinforcement.** Force–displacement curves obtained from displacement-controlled compression testing of bioinspired ring specimens, where the black curve corresponds to the ring containing approximately 10% continuous carbon fiber reinforcement and the red curve corresponds to the non-reinforced ring. Scale bar is 5mm.

References

1. Braga, G. C.; Couto, S. M.; Hara, T.; Almeida Neto, J. T. P. Mechanical behaviour of macadamia nut under compression loading. J. Agric. Eng. Res. 1999, 72, 239–245. <https://doi.org/10.1006/jaer.1998.0369>.
2. Khazaei, J.; Rasekh, M.; Borghei, A. M. Physical and mechanical properties of almond and its kernel related to cracking and peeling. ASAE Annual Meeting, 2002; Paper No. 026083.
3. Sharifian, F.; Derafshi, M. H. Mechanical behavior of walnut under cracking conditions. J. Appl. Sci. 2008, 8, 886–890. 886-890
4. Ojolo, J. S.; Eweina, B. A. Predicting cashew nut cracking using Hertz theory of contact stress. J. Saudi Soc. Agric. Sci. 2019, 18, 157–167.[https://doi.org/10.1016/j.jssas.2017.04.002](https://doi.org/10.1016/j.jssas.2017.04.002).
5. Güner, M.; Dursun, E.; Dursun, İ. G. Mechanical behaviour of hazelnut under compression loading. Biosyst. Eng. 2003, 85, 485–491. <https://doi.org/10.1016/S1537-5110(03)00089-8>.
6. Nazari Galedar, M.; Mohtasebi, S. S.; Tabatabaeefar, A.; Jafari, A.; Fadaei, H. Mechanical behavior of pistachio nut and its kernel under compression loading. J. Food Eng. 2009, 95, 499–504. <https://doi.org/10.1016/j.jfoodeng.2009.06.009>.
7. Işik, E.; Ünal, H. Moisture dependent physical properties of white speckled red kidney bean grains. J. Food Eng. 2007, 82, 209–216. <https://doi.org/10.1016/j.jfoodeng.2007.02.012>.
8. Teotia, M. S.; Ramakrishna, P.; Berry, S. K.; Kour, S. Some engineering properties of pumpkin (Cucurbita moschata) seeds. J. Food Eng. 1989, 9, 153–162.
